# Formation of HopQ1:14-3-3 complex in the host cytoplasm modulates nuclear import rate of *Pseudomonas syringae* effector in *Nicotiana benthamiana* cells

**DOI:** 10.3389/fpls.2024.1335830

**Published:** 2024-03-04

**Authors:** Wojciech Rymaszewski, Fabian Giska, Marcin A. Piechocki, Patrycja B. Zembek, Magdalena Krzymowska

**Affiliations:** Institute of Biochemistry and Biophysics, Polish Academy of Sciences, Warsaw, Poland

**Keywords:** *Pseudomonas syringae*, HopQ1, cellular trafficking, nuclear translocation signal, protein complexes

## Abstract

HopQ1, a type three effector from *Pseudomonas syringae* upon phosphorylation coopts plant 14-3-3 proteins to control its stability and subcellular localization. Mass spectrometry of the cytoplasm-restricted effector revealed that HopQ1 already in this subcellular compartment undergoes phosphorylation at serine 51 within the canonical 14-3-3 binding motif and within the second putative 14-3-3 binding site, _24_RTPSES_29_. Our analyses revealed that the stoichiometry of the HopQ1:14-3-3a complex is 1:2 indicating that both binding sites of HopQ1 are involved in the interaction. Notably, _24_RTPSES_29_ comprises a putative nuclear translocation signal (NTS). Although a peptide containing NTS mediates nuclear import of a Cargo protein suggesting its role in the nuclear trafficking of HopQ1, a deletion of _25_TPS_27_ does not change HopQ1 distribution. In contrast, elimination of 14-3-3 binding site, accelerates nuclear trafficking the effector. Collectively, we show that formation of the HopQ1:14-3-3 complex occurs in the host cytoplasm and slows down the effector translocation into the nucleus. These results provide a mechanism that maintains the proper nucleocytoplasmic partitioning of HopQ1, and at the same time is responsible for the relocation of 14-3-3s from the nucleus to cytoplasm in the presence of the effector.

## Introduction

*Pseudomonas syringae*, like many pathogenic gram-negative bacteria employs type three secretion system to deliver proteinaceous effectors directly into the cytosol of eukaryotic host cells. The effectors are involved in nutrient acquisition or suppression of defense response, and thereby facilitate successful colonization of plant tissues (Büttner, 2012). Various pathovars of *P. syringae* possess HopQ1 (for Hrp outer protein Q) in their repertoire of effectors. HopQ1 was shown to promote bacterial speck disease of tomato and halo blight of bean (Wei et al., 2007; Ferrante et al., 2009; Li et al., 2013a); but it is also produced by *P. syringae* strains identified as causal agents of recurring epidemics of chestnut or kiwifruit bleeding cankers (Green et al., 2010; Choi et al., 2017). Interestingly, our studies (Piechocki et al., 2018) revealed existence of putative HopQ1 homologs in other pathogenic bacteria and fungi but surprisingly also in lower plants and algae, including *Aureococcus anophagefferens* a dominant species responsible for destructive brown tide blooms (Gobler and Sunda, 2012). HopQ1 – like proteins (HLPs) show overall homology to nucleoside hydrolases, but due to alterations in the predicted catalytic center compared to the consensus sequence were classified as a distinct family (Piechocki et al., 2018). HopQ1 was shown to alter purine metabolism and activate cytokinin signaling (Li et al., 2013b; Hann et al., 2014) whereas recent studies revealed that XopQ, a HopQ1 homolog from *Xanthomonas euvesicatoria* exerts 2’,3’-cAMP/cGMP phosphodiesterase activity, which is possibly employed by bacteria to hydrolyze signaling molecules produced by a TIR domain of TNL subclass of plant resistance receptors (Yu et al., 2022).

Previous studies showed that HopQ1, as well as XopQ, after specific phosphorylation *in planta*, binds to host 14-3-3 proteins, a highly-conserved family of proteins that function as regulators of their partner proteins (Giska et al., 2013; Li et al., 2013a; Teper et al., 2014). The Interaction with 14-3-3 proteins affects subcellular distribution and stability of HopQ1 and thereby modulates virulence properties of the effector. The major goal of these studies was to further characterize the complex of HopQ1 protein with 14-3-3s in terms of its structure and spatial dynamics.

## Materials and methods

### Constructs for subcellular localization

To generate GUS-YFP fusion (Cargo) a sequence encoding *E. coli* K12 β-D-glucuronidase (*GUS, gusA*, formerly *uidA*) (Wilson et al., 1995) was PCR-amplified and cloned into the pENTR/D-TOPO vector (see **Supplementary Table S1**). The resulting entry clone was LR recombined with the pGWB 441 destination vector (Nakagawa et al., 2007; Nakagawa et al., 2009). To make HopQ1-Cargo construct, BamHI and XhoI restriction sites were introduced at 5’of *GUS* in the pENTR-Cargo plasmid. Next, the wild-type *hopQ1* sequence was cloned upstream of *GUS*. The resulting plasmid was LR recombined with the pGWB 441 destination vector.

To generate HopQ1-mDendra2 constructs, the sequence encoding mDendra2 was PCR-amplified with primers adding Eco47III-SacI restrictions sites (**Supplementary Table S1**), cloned into pJET1.2 and next re-cloned into pGWB 414 vector. The resulting plasmid was used for LR recombination.

Similarly, NTS-GUS-mDendra2 constructs were prepared, but the upper primers used for *GUS* amplification included variants of *hopQ1*- or *AtMPK6*-derived sequence encoding the 16-amino-acid fragment with wild-type NTS, phoshomimic (EPE) or phosphonull (APA) form (**Supplementary Table S1**). The resulting entry clone was LR recombined with pGWB 414 vector containing mDendra2.

### Confocal microscopy

The plasmids were electroporated into *Agrobacterium tumefaciens* (GV3101) cells, which were then infiltrated into leaves of 4-week-old *N. benthamiana* plants grown in soil under controlled environmental conditions (21°C, 16 h light, 8 h dark) as described previously (Giska et al., 2013; Zembek et al., 2018).

Transient intracellular fluorescence was observed using a NikonC1 confocal system configured on TE2000E inverted confocal microscope and equipped with 60x Plan-Apochromat oil immersion objective (Nikon Instuments B.V. Europe, Amsterdam, Netherlands). Fluorescence of eYFP and mDendra2 was excited with a Sapphire 488 nm laser and observed using 515/530 nm or 500/560 nm emission filter, respectively. Scanning was performed in sequential mode to prevent bleed-through. Images were collected in z- stack series at 0.5 μm focus interval. Single optical sections with distinctly visible nucleoli were selected to ensure that similar focal planes were compared for all tested variants. Quantification of fluorescence intensities in the nuclear and cytoplasmic regions was performed using ImageJ software (Abramoff et al., 2004).

### Photoconversion and image processing

Cimeric mDendra2 proteins were transiently expressed in *N. benthamiana* and evaluated using Olympus FV1000 confocal system (Olympus, Tokyo, Japan) built on a IX81 frame and equipped with 60 x UPlan-Apochromat water immersion objective. Fluorescence imaging was focused on the cellular region containing the nucleus for analysis of nuclear import and export of fusion proteins. mDendra2 was photoconverted by continuous illumination of a chosen region of interest (ROI) within the cytoplasm with 405 nm laser diode. For visualization of unconverted pool of protein, mDendra2 was excited with the 488 nm line of an argon-ion laser and observed in the 500-560 nm band. The photoconverted fraction was excited with a 559 nm laser diode and observed in the 570-640 nm band. The nuclear fluorescence intensity of converted and unconverted Dendra2 was quantified using Fiji (Schindelin et al., 2012). Data analyses were carried out in R (http://www.r-project.org). The fluorescence signal changed in a linear fashion within the first 80 seconds of photoconversion, so nuclear import and export rates were calculated by fitting a linear model.

### Protein expression and purification

Recombinant HopQ1 (AAZ37975.1) from *Pseudomonas syringae* pv. *phaseolicola* 1448A was produced in *Escherichia coli* BL21 Rosetta in fusion with 6xHis-tag and purified by affinity and ion-exchange chromatography (Q-Sepharose column; GE Healthcare, Chicago, US), as previously described (Giska et al., 2013). The recombinant Nt14-3-3a protein (BAD12168.1) tagged with Strep-tag II was produced in *E. coli* and purified by affinity and ion-exchange chromatography. To reconstitute the complex, first HopQ1 was *in vitro* phosphorylated by AtCPK3 (AEE84789.1) fused to GST-tag. To remove the AtCPK3 kinase, the reaction was loaded onto a GST-binding column. HopQ1 was further purified by ion-exchange and size-exclusion chromatography (Superdex 200 10/300GL; GE Healthcare, Chicago, US). Next, phosphorylated HopQ1 was mixed with Nt14-3-3a at a 1:2 mass ratio and incubated in buffer containing 20 mM Tris-HCl, 150 mM NaCl, and 5 mM DTT, pH 8.0 for 2 h at 4°C on a rotator.

To purify HopQ1-Cargo, the infiltrated tissue was ground in a mortar to a powder, which was suspended in buffer: 500 mM NaCl, 50 mM NaH_2_PO4, 10 mM imidazole, 5% [v/v] glycerol, 10% [v/v] protease inhibitor cocktail (Bioshop, Ontario, Canada), 10% [v/v] phosphatase inhibitor cocktail (Sigma-Aldrich, Burlington, US), 10 mM DTT, pH 8.0 and left on ice to thaw. The His-tagged GBP (GFP-binding protein) suspended in the same buffer was applied to a Ni-NTA column (ThermoScientific, Walthman, US). A plant extract was applied twice to the column prepared in the previous step and washed twice. Protein elution was performed twice with buffer: 500 mM NaCl, 50 mM NaH_2_PO4, 250 mM imidazole, 5% [v/v] glycerol, pH 8.0. The eluted protein fractions were combined and concentrated to a volume of ~100 µl, while replacing the buffer with the buffer: 10 mM Tris-HCl, 150 mM NaCl, pH 8.0. Samples (15 µl) were collected from each step and fractionated in a polyacrylamide gel, then the proteins were transferred to a membrane and detected using α-GFP-conjugated AP antibodies (Abcam, Walthman, US).

### Multi-angle light scattering analysis

For MALS samples were separated using Superdex 200 10/300GL column in buffer containing 100 mM Tris-HCl pH 8.0, 150 mM NaCl and 5 mM DTT. The analysis was performed using the Wyatt Dawn Heleos-II (apparatus) multi-angle bright scattering detector connected to Optilab T-rEX differential refractometer (Wyatt Technologies, Santa Barbara, USA). The data were analyzed using ASTRA 6.1 software.

### Statistical analyses

Statistical analyses were performed as follows: data were tested for normality of distribution by a Shapiro—Wilk test. If the data were suitable for conducting parametric tests then repeated-measures analysis of variance (ANOVA) was performed. For *post-hoc* testing, Tukey’s honestly significant difference (HSD), or Mann-Whitney tests were applied. Data are means from 2-4 independent experiments.

## Results and discussion

### HopQ1 phosphorylation and complex assembly with 14-3-3 occurs within the host cytoplasm

**Figure 1:**
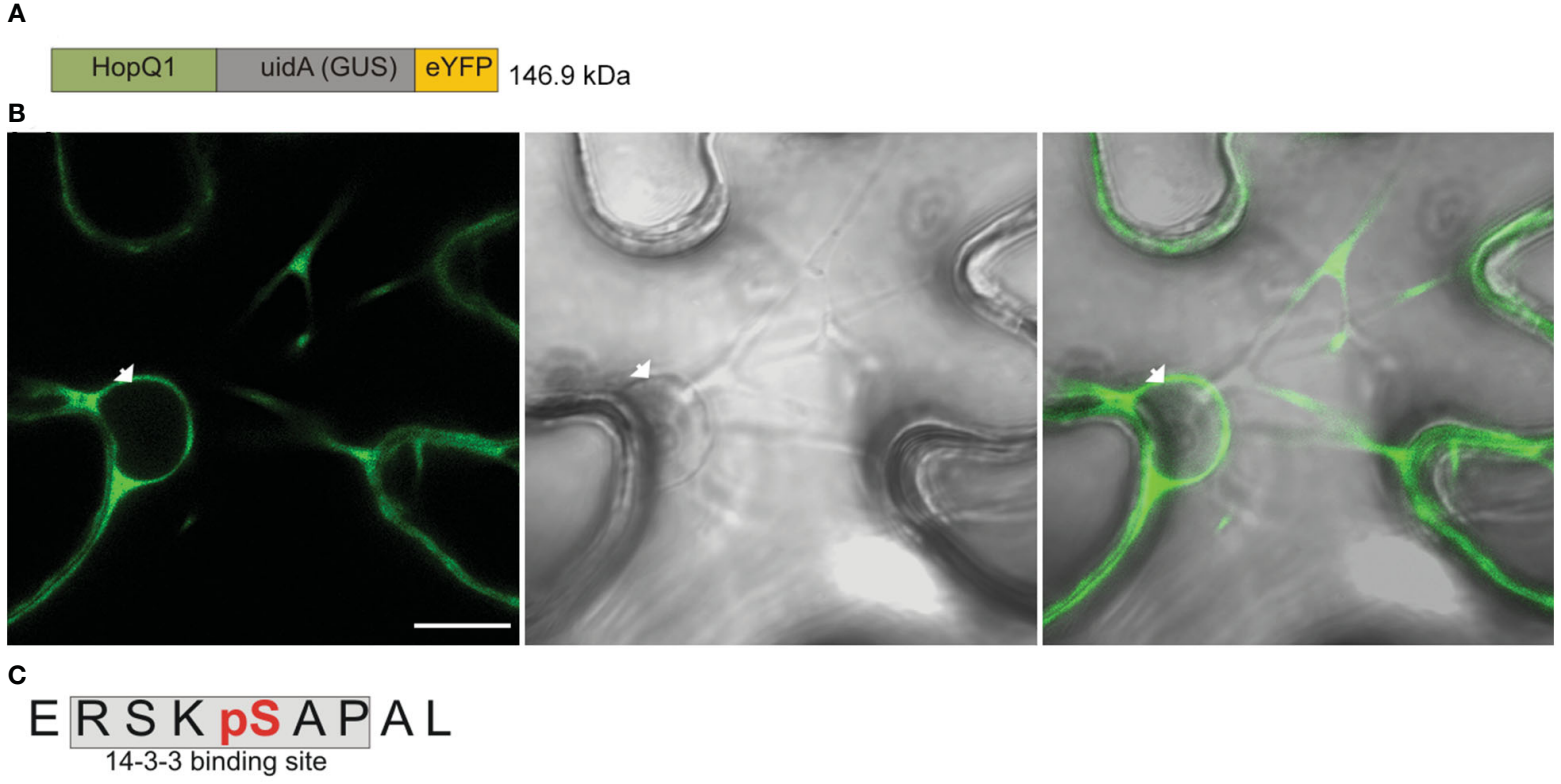
HopQ1 is phosphorylated in the host cytoplasm. **(A)** Schematic presentation of the chimeric HopQ1-Cargo protein, which consists of the effector fused to *E. coli* K12 β-D- glucuronidase (GUS) and yellow fluorescent protein (YFP). **(B)** Confocal image of the representative leaf epidermal cell transiently expressing HopQ1-Cargo taken 72 h after agroinfiltration. White arrowhead indicates the nucleus. Scale bar represents 10 μm. **(C)** Sequence of the phosphopeptide derived from the N-terminus of HopQ1-Cargo. The 14-3-3- interacting motif is shaded in gray, and the phospho-Ser, identified by mass spectrometry, is highlighted in red. Mascot analysis of this experiment is shown in **Supplementary Figure S2**.

The wild-type HopQ1 displays nucleocytoplasmic distribution, but it is predominantly localized to the cytoplasm of plant cells; whereas a variant of HopQ1 with an altered 14-3-3-binding site (HopQ1-S51A) is localized almost exclusively to the nucleus (Giska et al., 2013, see also **Supplementary Figure S1**). This finding raised two interlinked questions, that is 1) in which cellular compartment does HopQ1 associate with plant 14-3-3 protein?, and 2) where does HopQ1 phosphorylation, which is a prerequisite for the 14-3-3s’ binding, occur? To address these questions, we generated a construct expressing HopQ1 fused to a high molecular weight chimeric protein (hereinafter called Cargo, (**Figure 1A**) containing yellow fluorescent protein (YFP) and glucuronidase (GUS, *uidA*). The construct was transiently expressed in *Nicotiana benthamiana* leaves *via* agroinfiltration. After 3 days of incubation, we examined HopQ1-Cargo localization. As expected, the fusion with Cargo prevented nuclear translocation, and the effector was confined to the cytoplasm (**Figure 1B**). In a parallel experiment, HopQ1-Cargo was affinity purified using GFP-binding protein (GBP) (Rothbauer et al., 2008) and subjected to mass spectrometry analysis. The results obtained (**Figure 1C**, **Supplementary Figure S1**) revealed a phosphorylated serine (pS) in the peptide ERSKpSAPAL that corresponds to S51, the central serine within the 14-3-3-binding motif of HopQ1. We previously showed that S51 phosphorylation occurring in plant cells is indispensable for HopQ1 interaction with 14-3-3 proteins (Giska et al., 2013). Our results corroborate the previously reported data and indicate that the effector is phosphorylated in the host cytoplasm. This means that already in the cytoplasm HopQ1 acquires the ability to assemble into the complex with 14-3-3 proteins. Consistent with this model, the HopQ1-Cargo fusion protein co-purified with several 14-3-3 isoforms (**Supplementary Figure S3**).

### Stoichiometry of HopQ1:14-3-3a complex

**Figure 2:**
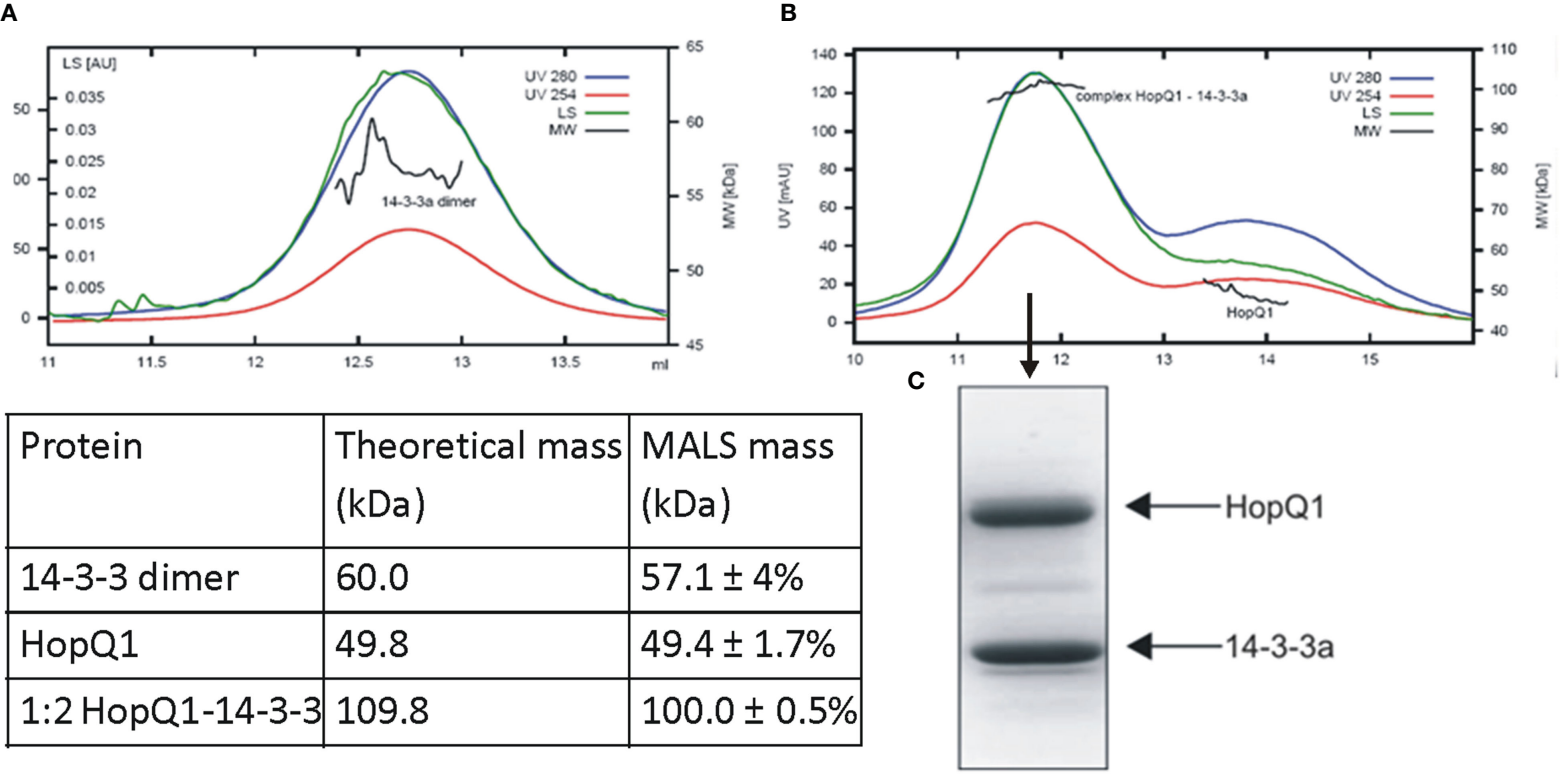
Stoichiometry of complex of HopQ1 with 14-3-3a. Recombinant 14-3-3a protein with a C-terminal Strep II epitope **(A)** and *in vitro* reconstituted HopQ1:14-3-3a complex **(B)** were subjected to gel filtration coupled with MALS analyses. Blue and red traces correspond to absorption at 280 nm and 254 nm, respectively; green traces indicate light scattering (LS) at 90° angle and black indicates molecular weights. Comparison of the theoretical molar masses for HopQ1, 14-3-3a and HopQ1:14-3-3a complex with the respective MALS derived molar masses is shown in the table. **(C)** Mid-peak fraction corresponding to HopQ1:14-3-3a complex was fractionated on 12.5% SDS-Page and stained with Coomassie Brilliant Blue.

Although 14-3-3s were among the first identified binding partners for HopQ1, the stoichiometry of the interaction remained unknown. To address this question, we performed size-exclusion chromatography coupled to MALS (Multi-Angle Light Scattering) analysis. This technique allows determination of molecular mass as a function of retention volume. First, the recombinant HopQ1-6xHis protein produced in *Escherichia coli* was *in vitro* phosphorylated by a recombinant AtCPK3 kinase, and upon incubation with Nt14-3-3a protein tagged with Strep-tag II, the mixture was subjected to the gel filtration followed by MALS. This analysis revealed that the stoichiometry of the HopQ1:14-3-3a complex is 1:2 that is one molecule of HopQ1 binds to a 14-3-3 dimer (**Figures 2A–C**, **Supplementary Figure S4**).

### N-terminal region of HopQ1 comprises at least two putative 14-3-3 binding site

Strikingly, the mass spectrometry analysis revealed also another series of phosphopeptides derived from the N-terminal region of HopQ1 (**Supplementary Figure S2**). They comprised a second putative 14-3-3 binding site, _24_RTPSES_29_, which does not conform either to mode 1 or mode 2. This site is located 19 aa apart from the previously characterized motif of HopQ1 containing S51. Notably, we have previously shown that 14-3-3 binding to the N-terminus of HopQ1 is mediated by S51 phosphorylation and S51A substitution completely abolished interaction between the effector and 14-3-3 protein. This fact and the close proximity between those two 14-3-3 binding motifs suggest that 14-3-3 dimer might bind two phosphoepitopes of HopQ1 cooperatively. It means that the interaction of one 14-3-3 subunit with HopQ1 *via* the dominant motif comprising S51 would facilitate binding of the second 14-3-3 subunit through the S27-containing motif. This suggests that both identified 14-3-3 binding sites are involved in the formation of the complex.

### Role of _24_RTPSES_29_ in the nuclear trafficking of HopQ1

**Figure 3:**
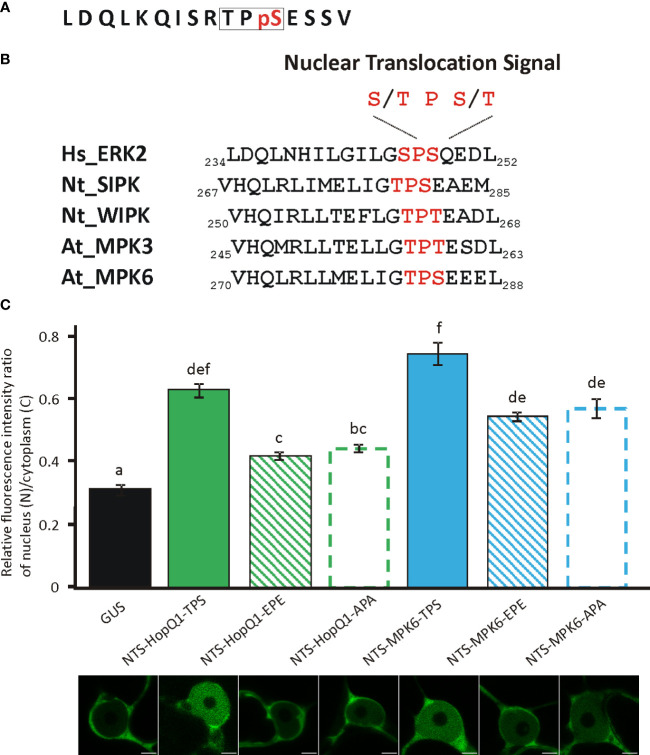
Functionality of NTS sequences and their phosphovariants from the HopQ1 and AtMPK6. **(A)** Amino acid sequence of the phosphopeptide comprising putative nuclear translocation signal (NTS) derived from HopQ1. NTS motif is framed and the phospho-Ser is highlighted in red. **(B)** Comparison of NTS surrounding from metazoan MAP kinases with the corresponding sequence identified in plant MAP kinases activated in immunity. NTS motif is highlighted in red. Hs *Homo sapiens*, Nt *N. benthamiana*, At *A. thaliana*. **(C)** The quantification of the ratio of the fluorescence intensity of nuclear and cytoplasmic fraction of mDendra2 in *N. benthamiana* epidermal leaf cells at 72 h after infiltration with *A. tumefaciens* strains carrying constructs indicated: 1) a control protein consisting of GUS and mDendra2, 2) wild-type NTS peptide derived from HopQ1 and 3) AtMPK6 (NTS-Q1-TPS, NTS-MPK6-TPS,respectively) 4) phosphomimic (EPE) or 5) phosphonull (APA) variants (NTS-Q1-EPE, NTS-Q1-APA, NTS-MPK6-EPE, NTS-MPK6-APA, respectively) fused to GUS- mDendra2. mDendra2 fluorescence intensity was measured for five areas within the nucleus and cytoplasm, and the average was calculated for each set of measurements. Then the ratio of average nuclear (N) to cytoplasmic (C) fluorescence was calculated for each tested cell. Error bars correspond to ± SEM. Letters indicate homogenous groups detected by one-way ANOVA and Tukey HSD *post hoc* test (p < 0.05).

Interestingly, the phosphopeptide _24_RTPSES_29_ contains also a putative nuclear translocation signal (NTS) reminiscent of the NTS motif identified in the metazoan MAP kinases (Chuderland et al., 2008). Phosphorylation of this motif (S/T-P-S/T) mediates translocation of those MAP kinases into the nucleus (Chuderland et al., 2008). Strikingly, this motif within a similar sequence context is present in plant MAP kinases (**Figure 3**) but its significance has not been experimentally validated. This finding is interesting in the light of the previous reports showing that HopQ1 and XopQ, its homolog from Xanthomonas spp. interfere with plant MAP kinase pathways (Hann et al., 2014; Teper et al., 2014; Zembek et al., 2018). Since the deletion of the TPS in HopQ1 sequence did not change the localization of the effector (**Supplementary Figure S5**), to test whether _25_TPS_27_ of HopQ1 (**Figure 3A**) as well as the corresponding motif of immune-activated MAPK6 from *Arabidopsis thaliana*, AtMAPK6 (_282_TPS_284_) (**Figure 3B**) may facilitate nuclear trafficking, we fused the peptides comprising NTS motifs to a high molecular weight chimeric protein, which consisted of GUS and Dendra2. Fusion of LDQLKQISRTPSESSV and VHQLRLLMELIGTPSEEEL, the peptides derived from HopQ1 and AtMPK6, respectively resulted in predominantly nuclear localization of the chimeric proteins (**Figure 3C**), suggesting that the wild-type variants of the peptides can efficiently drive protein translocation into the nucleus. To determine whether this process was dependent on the phosphorylation, we generated phosphovariants of the peptides by replacing TPS motif with a phosphonull (APA) or phosphomimic (EPE) tripeptide, and subsequently we tested whether the peptides mediated nuclear translocation of the reporter protein. Surprisingly, the fusion of either phosphomimic or phosphonull peptides resulted in a nucleocytoplasmic distribution pattern of the chimeric proteins but with a smaller nuclear pool than observed for the wild-type versions (**Figure 3C**). These findings corroborate the role of the peptides tested in the translocation to the nucleus meanwhile indicating that an efficient transport may require phosphorylation *per se*. Collectively, these results suggest that the _24_RTPSES_29_ motif might be involved not only in the interaction with 14-3-3s as the secondary binding site but also in facilitating nuclear import of HopQ1, thereby fine-tuning the effector’s subcellular distribution.

### Complex formation with 14-3-3s decelerates effector translocation into the nucleus

**Figure 4:**
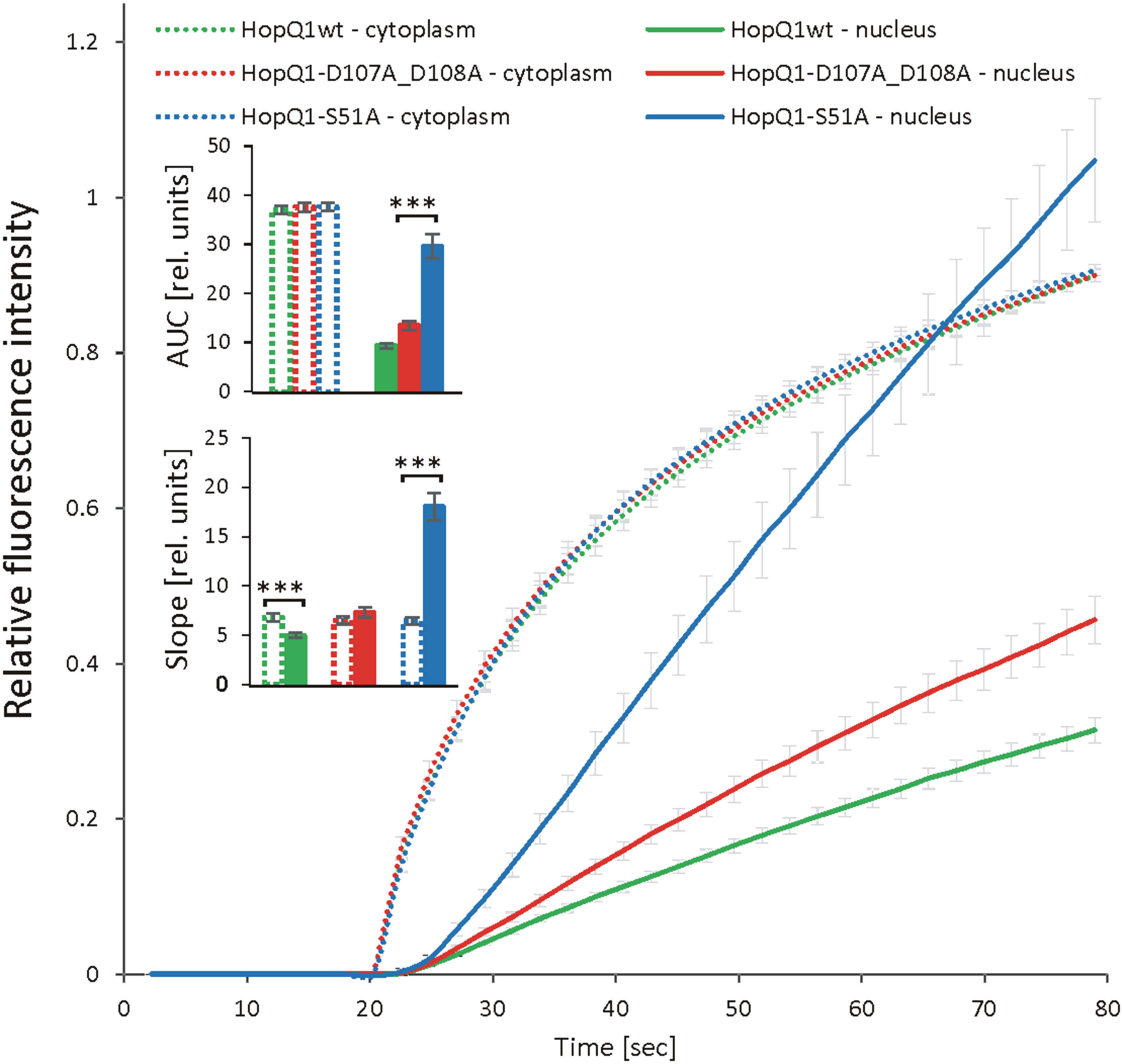
Dynamics of HopQ1 flow between the cytoplasm and nucleus. HopQ1 variants fused to photoconvertible mDendra2 protein were transiently expressed in *N. benthamiana* cells. Green-to-red photoconversion was achieved by laser stimulation of a defined spot in the cytoplasm from 20 sec of imaging onward. Red fluorescence intensity was recorded in the cytoplasm and nuclei of 50-70 cells per variant during 6 consecutive experiments. Curves for both measured cellular compartments show changes in red fluorescence intensities normalized by the final intensity in the cytoplasm. Areas under curves (AUCs) were analyzed statistically with an ANOVA and Tukey’s HSD test and the slope (starting with 50 s) with Mann-Whitney test (***p < 0.001).

To get more insight into the role of 14-3-3s in the cellular trafficking of HopQ1, we fused the wild-type HopQ1, HopQ1-S51A, the variant of the effector unable to bind 14-3-3s, as well as HopQ1-D107A_D108A, the variant where the aspartate motif’s DXXXDXDD predicted to bind calcium has been mutated (DXXXDXAA), to Dendra2. Dendra2 is a protein that undergoes photoconversion from green to red fluorescence (Gurskaya et al., 2006; Chudakov et al., 2007a; Chudakov et al., 2007b). This feature enables tracking of the activated form of the protein and its replacement by the non-activated form. The wild-type HopQ1-Dendra2 when transiently expressed in *N. benthamiana* displayed typical nucleocytoplasmic distribution. Subsequently, a selected small area within the cytoplasm was illuminated, and we monitored the flow of the chimeric protein within the cell. After illumination, HopQ1-Dendra2 in the photoconverted state moved immediately throughout the cytoplasm and was rapidly translocated to the nucleus (**Supplementary Movie S1**). Similarly, HopQ1-S51A variant was analyzed, and we compared changes in the nuclear and cytoplasmic fluorescence intensities of each fusion protein in the activated state (red fluorescence). As shown in **Figure 4** (see also **Supplementary Movie S1**), the rate of HopQ1-S51A translocation to the nucleus recorded as an increase in the nuclear fluorescence intensity of the photoconverted HopQ1-S51A-Dendra2 was constant and independent of its cytoplasmic level. Finally, such a linear trend led to the higher concentration of HopQ1-S51A-Dendra2 in the nucleus than in the cytoplasm. This indicates that HopQ1-S51A is actively transported to the nucleus. In contrast, a passive trafficking most possibly accounts for a relationship between the nuclear and cytoplasmic flow of the wild-type HopQ1. This indicates that the interaction of the effector with 14-3-3s slows down the HopQ1 nuclear import. Although, the interaction of HopQ1 with 14-3-3s possibly buries the NTS motif, this does not account for the observed difference between the translocation of HopQ1 and HopQ1-S51A, since the deletion of NTS in both variants does not change their distribution patterns (**Supplementary Figure S5**). In the experiment in **Figure 4**, we also included HopQ1-D107A_D108A, a variant mutated in the predicted calcium binding site, which displayed also accelerated nuclear translocation, compared to the wild-type HopQ1, implying that calcium may provide another regulatory level of HopQ1 trafficking. Collectively, our results show that interaction with 14-3-3s plays a critical role in determining nucleocytoplasmic equilibrium of HopQ1, however other factors might be involved in fine-tuning its distribution.

The function of the elements of MAP kinase cascades that play a role in the plant immunity depends on 14-3-3 proteins (Cooper et al., 2003; Oh et al., 2010; Oh and Martin, 2011; Dong et al., 2023). Their activity and stability is controlled by 14-3-3s. A virulence mechanism used by necroviruses relies upon competition among the viral coat protein and MAPKKKα for binding to 14-3-3s (Gao et al., 2022). Outcompeting of 14-3-3s leads to destabilization of the kinase. We showed that the interaction of HopQ1 with 14-3-3s reciprocally affects subcellular localization of both partners. Co-expression of HopQ1, but not HopQ1-S51A, with 14-3-3a leads to relocation of 14-3-3a from the nucleus to the cytoplasm (Giska et al., 2013). Our data suggest that employment of 14-3-3s by bacteria serves to dynamically control HopQ1 nucleocytoplasmic equilibrium, maintaining the major pool of the effector in the cytoplasm. Importantly, the both subpools of HopQ1 are required to promote bacterial growth in plants (**Supplementary Figure S6**). On the other hand, we cannot exclude that this strategy serves also to capture 14-3-3s in the cytoplasm resulting in the destabilization of plant proteins, including MAP kinases. In summary, HopQ1, the effector that interferes with plant MAP kinase pathway co-opts host machinery controlling this pathway such as 14-3-3s and NTS to regulate its own localization and stability.

## Data availability statement

The original contributions presented in the study are included in the article/**Supplementary Material**. Further inquiries can be directed to the corresponding author.

## Author contributions

WR: Conceptualization, Investigation, Methodology, Writing – review & editing, Visualization. FG: Conceptualization, Investigation, Writing – review & editing. MP: Investigation, Writing – review & editing. PZ: Visualization, Writing – review & editing. MK: Conceptualization, Funding acquisition, Project administration, Supervision, Writing – original draft.

## Supplementary material

Supplementary Movie S1 HopQ1 and HopQ1-S51A trafficking within the host cell.
