# Supplementary material for: Formation of HopQ1:14-3-3 complex in the host cytoplasm modulates nuclear import rate of Pseudomonas syringae effector in Nicotiana benthamiana cells
Source: Front Plant Sci. 2024 Mar 4;15:1335830. doi: 10.3389/fpls.2024.1335830 (PMC10944878; doi:10.3389/fpls.2024.1335830)
Supplement: Supplementary file 2 [file DataSheet_1.pdf]

Table S1 Primers used in this study.

Name	Sequence	Task
**GW-uidA-F**	CACCATGTTACGTCCTGTAGAAACCCCAAC	**Cloning of glucuronidase coding sequence (*uida*, *gus*) to pENTR D-TOPO**
**GW-uidA-R**	TTGTTTGCCTCCCTGCTG	
**GUS-NTS-WT-F**	CACCATGGACCTAGATCAGTTAAAACAAATAT CACGTACCCCATCGGAAAGTAGCGTGATGTTA CGTCCTGTAGAAACC	**Cloning of putative NTS from *HopQ1–CARGO* to pENTR D-TOPO**
**GUS-NTS-EPE-F**	CACCATGGACCTAGATCAGTTAAAACAAATAT CACGTGAACCAGAAGAAAGTAGCGTGATGTTA CGTCCTGTAGAAACC	
**GUS-NTS-APA-F**	CACCATGGACCTAGATCAGTTAAAACAAATAT CACGTGCACCAGCAGAAAGTAGCGTGATGTTA CGTCCTGTAGAAACC	
**GUS-NTS-APE-F**	CACCATGGACCTAGATCAGTTAAAACAAATAT CACGTGCACCAGAAGAAAGTAGCGTGATGTTA CGTCCTGTAGAAACC	
**GUS-NTS-EPA-F**	CACCATGGACCTAGATCAGTTAAAACAAATAT CACGTGAACCAGCAGAAAGTAGCGTGATGTTA CGTCCTGTAGAAACC	
**Gus2022d**	CTGGACTGGCATGAACTTCGGTGA	**Sequencing of *CARGO***
**T-gus-bx-F**	GGATCCCTCGAGTTACGTCCTGTAGAAACC	**Adding of BamHI and XhoI restriction sites to pENTR D-TOPO CARGO**
**T-gus-bx-R**	CATGGTGAAGGGGGCGGCCGCGGAG	
**NTS-MPK6-TPS-F**	CACCATGGTCCATCAGCTTCGCTTGCTCATGGA GCTCATAGGAACTCCATCAGAAGAAGAGCTCA TGTTACGTCCTGTAGAAACCCCA	**Cloning of putative NTS from *AtMPK6–CARGO* to pENTR D-TOPO**
**NTS-MPK6-EPE-F**	CACCATGGTCCATCAGCTTCGCTTGCTCATGGA GCTCATAGGAGAGCCAGAGGAAGAAGAGCTCA TGTTACGTCCTGTAGAAACCCCA	
**NTS-MPK6-APA-F**	CACCATGGTCCATCAGCTTCGCTTGCTCATGGA GCTCATAGGAGCTCCAGCAGAAGAAGAGCTCA TGTTACGTCCTGTAGAAACCCCA	
**GW-Dendra-Eco47III**	GTGCAGCGCTATGAGAGGATCGGGATCCATGAAC	**Cloning Dendra2 coding sequence into pGWB vectors**
**GW-Dendra-SacI**	ATTCGAGCTCTCACTTGTACACGCCGCTGTC	
**HopQ1DD-F**	AAGGACCCGGCTGCTGTCGTGACCTACACCTTG	**Making of HopQ1-D107A_D108A mutant**
**HopQ1DD-R**	GTTAGGATCAGTGAAAAACCACGTATCCTTAG	
