# Supplementary material for: Formation of HopQ1:14-3-3 complex in the host cytoplasm modulates nuclear import rate of Pseudomonas syringae effector in Nicotiana benthamiana cells
Source: Front Plant Sci. 2024 Mar 4;15:1335830. doi: 10.3389/fpls.2024.1335830 (PMC10944878; doi:10.3389/fpls.2024.1335830)
Supplement: Supplementary file 3 [file DataSheet_2.pdf]

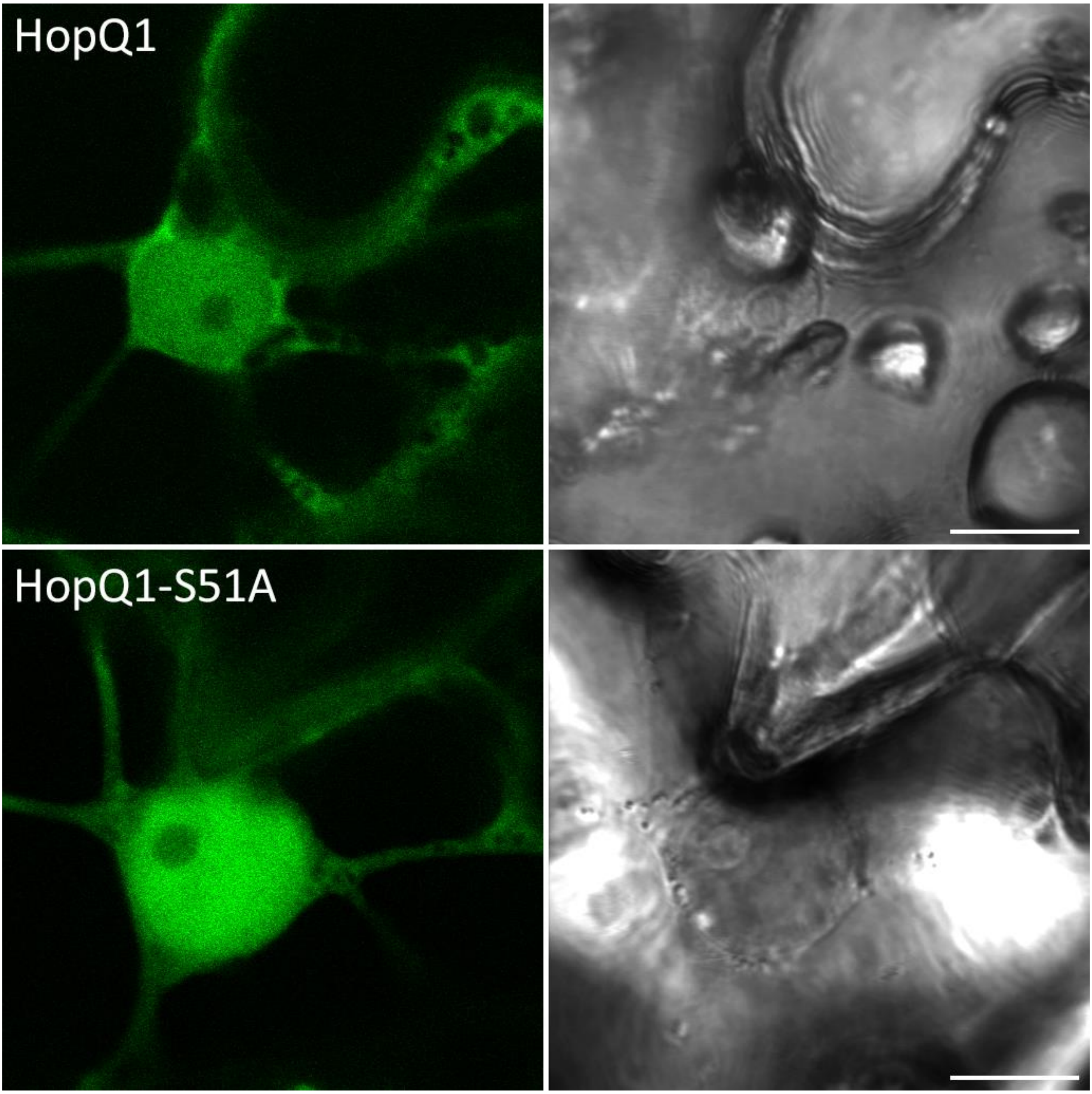

FIGURE S1 Subcellular distribution of HopQ1 and HopQ1_S51A variant.

MASCOT Search Results

Protein View: 121

HopQ1-GUS-eYFP

**Database:**	small-www
**Score:**	2121
**Nominal mass (M_r):**	146937
**Calculated pI:**	5.68

Sequence similarity is available as an NCBI BLAST search of 121 against nr.

Search parameters

**MS data file:**	\\212.87.29.88\Orbita\MI\404181059krzy_CARGO_peps.raw
**Enzyme:**	No enzyme cleavage specificity.
**Fixed modifications:**	Methylthio (C)
**Variable modifications:**	Oxidation (M), Phospho (ST), Phospho (Y)

Protein sequence coverage: 20%

Matched peptides shown in ***bold red***.

1	MGSMHRPITA	GHTTSRL**ILD**	**QLKQISRTPS**	**ESSVQ**SALSQ	QASMSSPVL**E**
51	**RSKSAPAL**LT	AAQRTMLAQV	GACNSHLTSD	ENMAINELRL	HKPRLPKDTW
101	F**FTDPNKDPD**	**DVVTYTLGK**Q	LQAEG**FVHIT**	**DVVATLGDAE**	**VRSQ**RAEMAK
151	GVFNKLGLHD	VHVSRGRDYA	MNSLQSKEHA	KF**LLEGHALR**	**AGPGEIHRD**S
201	LQDMSRRLAR	APHGVGIVVI	AGMSDINALI	TTCPDMVRER	VDDITIMGGV
251	EPLKDADGFV	QPDARAYNNA	TDMDAARSLY	RKAQELGIPL	RIVTKEAAYK
301	TAVSPSF**YEG**	**IAGSGHPVGH**	YLRDVQKSAL	KGLWEGIQAG	LLPGLDDSWF
351	FRTFMPNAQI	EAAQLDKNKE	SS**FEDIWPK**V	TKLNLYDPLT	L**LASVPGAAK**
401	**LL**FKPKA**IHT**	**EGFGVVEQVG**	**PDDVTHPE**KA	KLLMSALAKS	AL**VQSTVAPD**
451	**LELRPVETPT**	**RE**IKKLDGLW	AFSLDRENCG	IDQRWWESAL	QESRAIAVPG
501	SFNDQFADAD	IRNYAGNVWY	QREVFIPKGW	AGQRIVLRFD	AVTHYGKVWV
551	NNQEVMEHQG	GYTPFEADVT	PYVIAGKSVR	ITVCVNNELN	WQTIPPGMVI
601	TDENGKKKQS	YFHDFFNYAG	IHRSVMLYTT	PNTWVDDITV	VTHVAQDCNH
651	ASVDWQVVAN	GDVSVE**LRDA**	**DQQVVATGQG**	**TSGTL**QVVNP	HLWQPGEGYL
701	YELCVTAKSQ	TECDIYPLRV	GIRSVAVKGE	QFLINHKPFY	FTG**FGRHEDA**
751	**DLRGK**GFDNV	LMVHDHALMD	WIGANSYRTS	HYPYAEEMLD	WADEHGIVVI
801	DETAAVGFNL	SLG**IGFEAGN**	**KPKEL**YSEEA	VNGETQQAHL	QAIKELIARD
851	KNHPSVVM**WS**	**IANEPDTRPQ**	GAREYFAPLA	EATRKLDPTR	PITCVNVMFC
901	DAHTDTISDL	FDVLCLNRYY	GWYVQSGDLE	TAEKVLEKEL	LAWQEKLHQP
951	IIITEYGVDT	LAGLHSMYTD	MWSEEYQCAW	LDMYHRVFDR	VSAVVGEQVW
1001	NFADFATSQG	ILRVGGNKKG	IFTRDRKPKS	AAFLLQKRWT	GMN**FGEKPQQ**
1051	**GGKQKGG**RAD	PAFLYKVVDN	SMVSKGEELF	TGVVPIL**VEL**	**DGDVNGHK**FS
1101	VSGEGEGDAT	YGKLTLKFIC	TTGKLPVPWP	TLVTTFGYGL	QC**FARYPDHM**
1151	KQHDFFKSAM	PEGYVQERTI	FFKDDGNYKT	RAEVKFEGDT	LVNRIELKGI
1201	DFKEDGNILG	HKLEYNYNSH	NVYIMADKQK	NGIKVNF**KIR**	**HNIEDGSVQL**
1251	**ADHYQQNTPI**	**GDGPVLLPDN**	**H**YLSYQSALS	KDPNEKRDHM	VLLEFVTAAG
1301	ITLGMDELYK	A			

Query	Start - End	Observed	Mr(expt)	Mr(calc)	ppm	M	Score	Expect	Rank	U	Peptide
8090	18 - 33	941.4773	1880.9401	1880.9244	8.34	0	44	0.0014	1	U	L.ILDQLKQISRTPSESS.V + Phospho (ST)
9358	18 - 35	703.6930	2108.0573	2108.0514	2.76	0	71	3.6e-06	1	U	L.ILDQLKQISRTPSESSVQ.S + Phospho (ST)
4777	23 - 35	763.8644	1525.7142	1525.7137	0.31	0	62	2.1e-05	1	U	L.KQISRTPSESSVQ.S + Phospho (ST)
2411	25 - 35	595.8036	1189.5926	1189.5939	-1.03	0	57	4.5e-05	1	U	Q.ISRTPSESSVQ.S
1311	50 - 58	519.7525	1037.4904	1037.4906	-0.20	0	38	0.0029	1	U	L.ERSKSAPAL.L + Phospho (ST)
1380	102 - 110	524.7317	1047.4489	1047.4509	-1.91	0	53	0.0001	1	U	F.FTDPNKDPD.D
5846	102 - 115	813.3732	1624.7318	1624.7257	3.75	0	51	0.00028	1	U	F.FTDPNKDPDDVVTY.T
4804	107 - 119	765.8628	1529.7111	1529.7015	6.33	0	34	0.013	1	U	N.KDPDDVVTYTLGK.Q + Phospho (ST)
79	126 - 131	366.1888	730.3630	730.3650	-2.79	0	34	0.0054	1	U	G.FVHITD.V
1891	132 - 142	565.3137	1128.6129	1128.6139	-0.91	0	87	2.4e-08	1	U	D.VVATLGDAEVR.S
3625	132 - 144	672.8654	1343.7163	1343.7045	8.80	0	37	0.0032	1	U	D.VVATLGDAEVRSQ.R
5341	183 - 197	523.9541	1568.8404	1568.8423	-1.24	0	52	0.00012	1	U	F.LLEGHALRAGPGEIH.R
2613	189 - 199	305.9174	1219.6405	1219.6422	-1.39	0	48	0.00027	1	U	A.LRAGPGEIHRD.S
2614	189 - 199	407.5553	1219.6440	1219.6422	1.50	0	38	0.003	1	U	A.LRAGPGEIHRD.S
1971	308 - 319	572.2749	1142.5352	1142.5356	-0.40	0	76	4.7e-07	1	U	F.YEGIAGSGHPVG.H
3125	308 - 320	427.5385	1279.5938	1279.5946	-0.62	0	91	2.4e-08	1	U	F.YEGIAGSGHPVGH.Y
3126	308 - 320	640.8048	1279.5950	1279.5946	0.32	0	101	2.2e-09	1	U	F.YEGIAGSGHPVGH.Y
764	373 - 379	467.7375	933.4604	933.4596	0.81	0	46	0.00039	1	U	S.FEDIWPK.V
738	392 - 401	463.7880	925.5614	925.5596	1.86	0	60	3.6e-06	1	U	L.LASVPGAAKL.L
2575	408 - 418	608.3036	1214.5926	1214.5932	-0.46	0	101	2.1e-09	1	U	A.IHTEGFGVVEQ.V
2576	408 - 418	608.3040	1214.5934	1214.5932	0.16	0	64	9.1e-06	1	U	A.IHTEGFGVVEQ.V
9986	408 - 428	754.6940	2261.0601	2261.0601	-0.016	0	89	7.5e-08	1	U	A.IHTEGFGVVEQVGPDDVTHPE.K
9987	408 - 428	754.6950	2261.0631	2261.0601	1.31	0	99	7.6e-09	1	U	A.IHTEGFGVVEQVGPDDVTHPE.K
6989	412 - 428	891.4235	1780.8324	1780.8268	3.15	0	79	4.8e-07	1	U	E.GFGVVEQVGPDDVTHPE.K
6733	413 - 428	862.9133	1723.8120	1723.8054	3.83	0	90	3.9e-08	1	U	G.FGVVEQVGPDDVTHPE.K
5369	414 - 428	789.3749	1576.7352	1576.7370	-1.11	0	67	6.6e-06	1	U	F.GVVEQVGPDDVTHPE.K
4752	415 - 428	760.8656	1519.7167	1519.7155	0.82	0	90	3.1e-08	1	U	G.VVEQVGPDDVTHPE.K

Query	Start - End	Observed	Mr(expt)	Mr(calc)	ppm	M	Score	Expect	Rank	U	Peptide
2431	418 - 428	597.2734	1192.5323	1192.5361	-3.18	0	52	0.00014	1	U	E.QVGPDDVTHPE.K
1493	419 - 428	533.2444	1064.4741	1064.4775	-3.13	0	73	1.1e-06	1	U	Q.VGPDDVTHPE.K
4767	443 - 456	762.4265	1522.8384	1522.8355	1.88	0	69	1.6e-06	1	U	L.VQSTVAPDLELRPV.E
9908	443 - 462	746.3993	2236.1759	2236.1699	2.68	0	73	1.3e-06	1	U	L.VQSTVAPDLELRPVETPTRE.I
8221	667 - 685	958.9891	1915.9637	1915.9600	1.97	0	98	5.4e-09	1	U	E.LRDADQQVVATGQGTSGTL.Q
7235	668 - 685	902.4506	1802.8866	1802.8759	5.94	0	112	2.2e-10	1	U	L.RDADQQVVATGQGTSGTL.Q
4014	744 - 755	350.9313	1399.6960	1399.6956	0.23	0	43	0.0012	1	U	G.FGRHEDADLRGK.G
3333	814 - 825	434.9067	1301.6982	1301.6979	0.18	0	61	1.3e-05	1	U	G.IGFEAGNKPKEL.Y
4080	859 - 870	707.3390	1412.6634	1412.6684	-3.54	0	73	1.3e-06	1	U	M.WSIANEPDTRPQ.G
4295	1044 - 1057	362.1922	1444.7396	1444.7423	-1.87	0	41	0.0019	1	U	N.FGEKPQQGGKQKGG.R
4296	1044 - 1057	482.5872	1444.7398	1444.7423	-1.71	0	66	5.2e-06	1	U	N.FGEKPQQGGKQKGG.R
4297	1044 - 1057	723.3783	1444.7420	1444.7423	-0.16	0	91	1.6e-08	1	U	N.FGEKPQQGGKQKGG.R
2343	1088 - 1098	394.8631	1181.5676	1181.5677	-0.094	0	75	6.8e-07	1	U	L.VELDGDVNGHK.F
1299	1143 - 1150	346.1599	1035.4580	1035.4596	-1.58	0	49	0.00024	1	U	C.FARYPDHM.K
3979	1238 - 1249	465.9154	1394.7243	1394.7266	-1.69	0	57	3.5e-05	1	U	F.KIRHNIEDGSVQ.L
3980	1238 - 1249	698.3706	1394.7267	1394.7266	0.086	0	78	2.5e-07	1	U	F.KIRHNIEDGSVQ.L
4711	1238 - 1250	503.6102	1507.8088	1507.8107	-1.22	0	76	3.1e-07	1	U	F.KIRHNIEDGSVQL.A
4712	1238 - 1250	754.9126	1507.8106	1507.8107	-0.067	0	86	3e-08	1	U	F.KIRHNIEDGSVQL.A
8701	1251 - 1269	1025.4999	2048.9852	2048.9803	2.39	0	125	1.4e-11	1	U	L.ADHYQQNTPIGDGPVLLPD.N
10054	1251 - 1271	767.7014	2300.0823	2300.0822	0.069	0	77	1.3e-06	1	U	L.ADHYQQNTPIGDGPVLLPDNH.Y

FIGURE S2 Cytoplasmic variant of HopQ1 is phosphorylated at two positions. HopQ1-GUS-eYFP fusion protein expressed in *Nicotiana benthamiana* leaves was affinity purified, digested with pepsin, and subjected to LC-MS/MS analyses. The MS data were searched against a database containing the sequence of chimeric HopQ1-GUS-eYFP protein. Search parameters included no enzyme cleavage specificity, Cys carbamidomethyl fixed modification, and variable modifications including Met oxidation and phosphorylation of Ser, Thr, or Tyr residues.

Mascot Search Results

Peptide Summary Report of selected protein hits

O49997 **Mass:** 30643 **Score:** 216 **Matches:** 4(4) **Sequences:** 3(3) **emPAI:** 0.36

14-3-3-like protein E OS=Nicotiana tabacum PE=1 SV=1

Query	Observed	Mr(expt)	Mr(calc)	ppm	Miss	Score	Expect	Rank	Unique	Peptide
87	**385.7072**	**769.3999**	**769.4010**	**-1.41**	**0**	**36**	**0.048**	**1**		**R.YLAEFK.T**
188	**408.7161**	**815.4176**	**815.4137**	**4.75**	**0**	**44**	**0.021**	**1**		**K.LAEQAER.Y**
10113	**823.7647**	**2468.2723**	**2468.2654**	**2.78**	**0**	**(79)**	**3.2e-06**	**1**	**U**	**K.ICDGILNLLESHLIPVASTAESK.V**
10114	**823.7653**	**2468.2740**	**2468.2654**	**3.48**	**0**	**168**	**3.8e-15**	**1**	**U**	**K.ICDGILNLLESHLIPVASTAESK.V**

P93214 **Mass:** 29551 **Score:** 80 **Matches:** 4(4) **Sequences:** 4(4) **emPAI:** 0.53

14-3-3 protein 9 OS=Solanum lycopersicum GN=TFT9 PE=2 SV=2

Query	Observed	Mr(expt)	Mr(calc)	ppm	Miss	Score	Expect	Rank	Unique	Peptide
87	385.7072	769.3999	769.4010	-1.41	0	36	0.048	1		R.YLAEFK.A
188	408.7161	815.4176	815.4137	4.75	0	44	0.021	1		K.LAEQAER.Y
412	**447.2595**	**892.5044**	**892.5018**	**2.94**	**0**	**72**	**1.7e-05**	**1**	**U**	**R.NLLSVGYK.N**
1998	**627.8302**	**1253.6458**	**1253.6404**	**4.27**	**1**	**40**	**0.032**	**1**	**U**	**K.ERENFVYVAK.L**

P93343 **Mass:** 29438 **Score:** 50 **Matches:** 3(3) **Sequences:** 3(3) **emPAI:** 0.38

14-3-3-like protein C OS=Nicotiana tabacum PE=1 SV=1

Query	Observed	Mr(expt)	Mr(calc)	ppm	Miss	Score	Expect	Rank	Unique	Peptide
87	385.7072	769.3999	769.4010	-1.41	0	36	0.048	1		R.YLAEFK.T
188	408.7161	815.4176	815.4137	4.75	0	44	0.021	1		K.LAEQAER.Y
1625	**592.2961**	**1182.5777**	**1182.5768**	**0.74**	**0**	**38**	**0.051**	**1**	**U**	**K.EAAESTLTAYK.A**

FIGURE S3 Cytoplasmic variant of HopQ1 co-purifies with 14-3-3s. HopQ1-GUS-eYFP fusion protein expressed in *Nicotiana benthamiana* leaves was affinity purified, digested with pepsin, and subjected to LC-MS/MS analyses. Search parameters included no enzyme cleavage specificity, Cys carbamidomethyl fixed modification, and variable modifications including Met oxidation and phosphorylation of Ser, Thr, or Tyr residues.

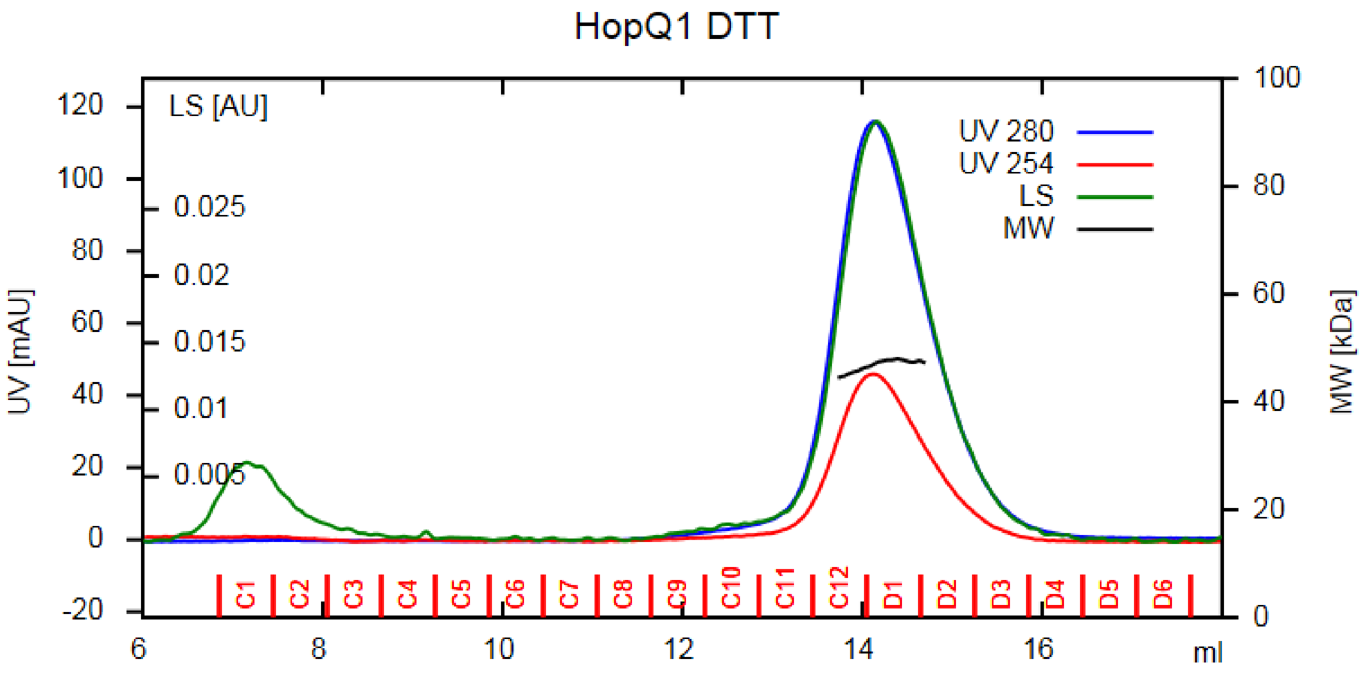

FIGURE S4 MALS analysis of HopQ1. Recombinant HopQ1 with a C-terminal 6xHis epitope was subjected to gel filtration coupled to MALS analysis in the presence of 5 mM DTT. Blue and red traces correspond to absorption at 280 nm and 254 nm, respectively; green trace indicates light scattering (LS) at 90° angle and black indicates molecular weight. The derived molar mass of HopQ1 is 46.8 kDa.

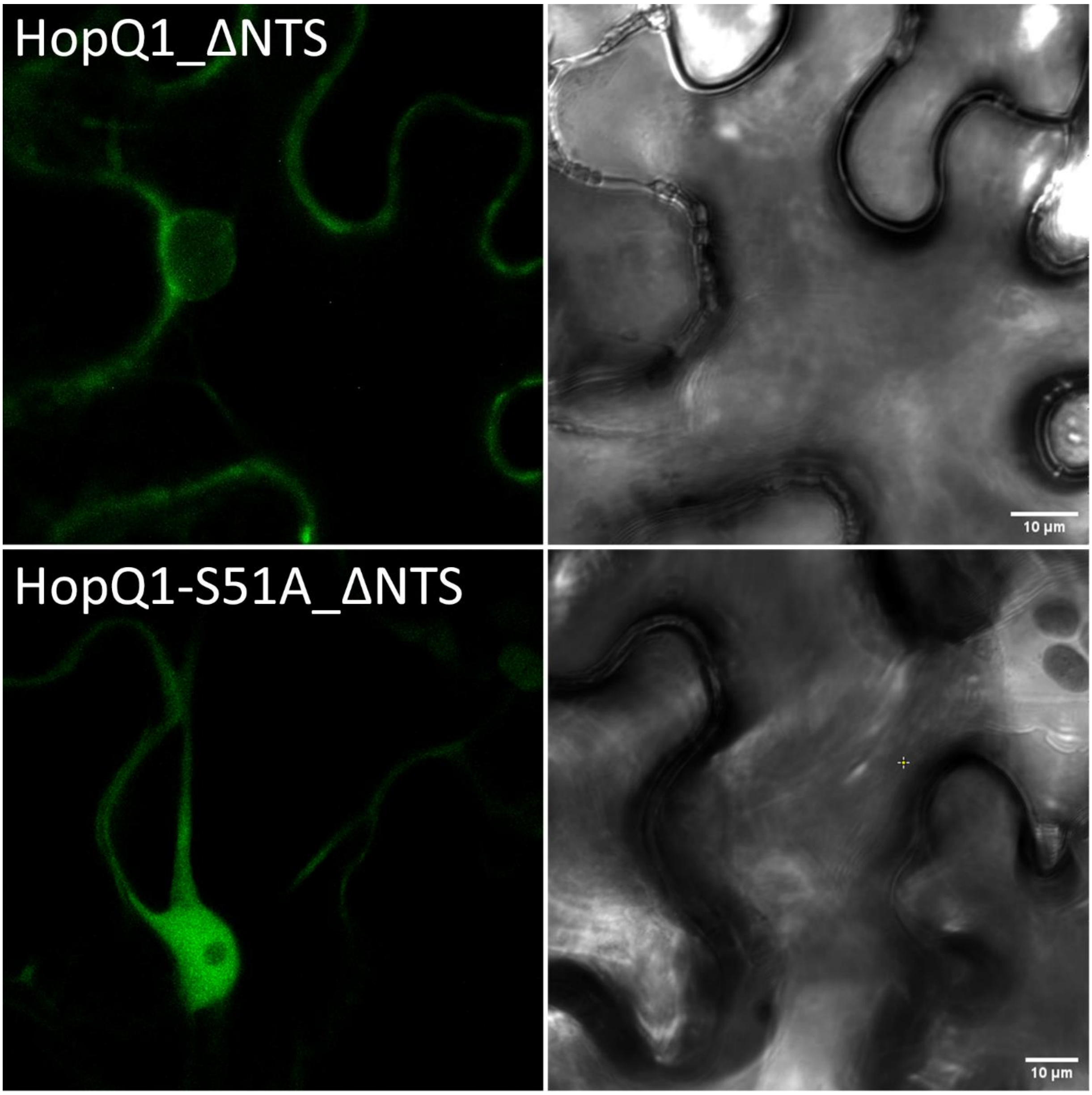

FIGURE S5 Deletion of NTS signal does not change HopQ1 partitioning. Fluorescence intensities in the nucleus and cytoplasm were determined by ImageJ software. The relative nuclear fractions for HopQ1_ΔNTS and HopQ1-S51A_ΔNTS, calculated as a ratio of the fluorescence intensity in the nucleus to the total fluorescence intensity in the cell, were 52 % and 72%, respectively. The equilibrium of HopQ1-S51A_ΔNTS distribution is shifted towards the nucleus, whereas the localization pattern of HopQ1_ΔNTS suggests that partitioning of HopQ1 variants is established by active and passive trafficking, respectively.

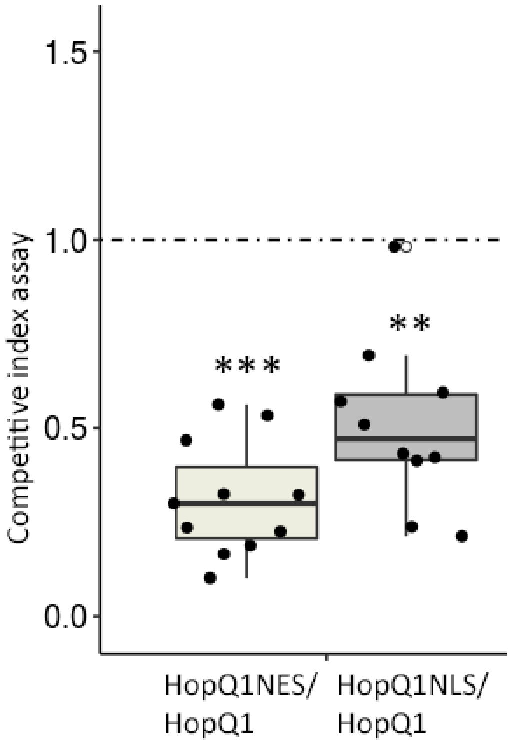

FIGURE S6 Nuclear and cytoplasmic pools of HopQ1 are indispensable to promote bacterial growth in bean plants. Strains of *Pseudomonas syringae* pv. *tomato* DC3000Δ28 (deficient in 28 effectors) carrying derivative of pBBR1MCS2 (Km^R) plasmid expressing HopQ1 fused either to nuclear localization signal (NLS) or nuclear export signal (NES) were mixed in a 1:1 ratio with the strain carrying pBBR1MCS5 (Gm^R) plasmid encoding the wild-type HopQ1. The mixed inocula were infiltrated into leaves of *Phaseolus vulgaris* cv. Red Mexican. Two days after inoculation tissue samples were collected, homogenized and plated onto LB medium, as described earlier by Giska et al. (2013). Next, the colonies obtained were replicated onto selective media to differentiate the strains. Competitive Index (CI) was calculated as the ratio of bacteria secreting the HopQ1 localization variants to bacteria secreting the wild-type HopQ1, normalized for bacterial load. Wilcoxon test was employed to establish whether CI was significantly different from 1 (** = $p<0.01$; *** = $p<0.001$). The experiment was repeated three times with similar results.
